# Time to gain trust and change—Experiences of attachment and mindfulness-based cognitive therapy among patients with chronic pain and psychiatric co-morbidity

**DOI:** 10.3402/qhw.v9.24420

**Published:** 2014-08-18

**Authors:** Birgitta Peilot, Paulin Andréll, Anita Samuelsson, Clas Mannheimer, Ann Frodi, Annelie J. Sundler

**Affiliations:** 1 Department of Molecular and Clinical Medicine/Multidisciplinary Pain Center, Institute of Medicine, Sahlgrenska Academy at the University of Gothenburg, Sahlgrenska University Hospital, Gothenburg, Sweden; 2 Health Centre, Landvetter, Sweden; 3 Developmental Psychology, University of Wisconsin, Iowa, Michigan, Rochester, NY, USA; 4 School of Health and education, University of Skövde, Skövde, Sweden; 5 Post Doctoral Research Fellow at School of Health, Care and Social Welfare, Mälardalens University, Västerås, Sweden

**Keywords:** Phenomenology, lived experiences, lifeworld, cognitive therapy, attachment, mindfulness, chronic pain, psychosocial issues

## Abstract

The treatment of patients with chronic pain disorders is complex. In the rehabilitation of these patients, coping with chronic pain is seen as important. The aim of this study was to explore the meaning of attachment and mindfulness-based cognitive therapy (CT) among patients with chronic pain and psychiatric co-morbidity. A phenomenological approach within a lifeworld perspective was used. In total, 10 patients were interviewed after completion of 7- to 13-month therapy. The findings reveal that the therapy and the process of interaction with the therapist were meaningful for the patients’ well-being and for a better management of pain. During the therapy, the patients were able to initiate a movement of change. Thus, CT with focus on attachment and mindfulness seems to be of value for these patients. The therapy used in this study was adjusted to the patients’ special needs, and a trained psychotherapist with a special knowledge of patients with chronic pain might be required.

The treatment of patients with chronic pain disorders is complex (Steihag, Ahlsen, & Malterud, 2001). Even if the group of patients with chronic pain have an identical medical diagnosis, these patients are not a homogeneous group, and they will not benefit equally from the same treatment (Turk, 2005; Turk, Okifuji, Siclair, & Starz, 1996). Living with chronic pain such as fibromyalgia can be a burden, restricting everyday life. In patients with chronic pain, impaired quality of life has been reported (Peilot, Andréll P, Mannerkorpi, & Mannheimer, 2010). Living with pain has also been described as unpredictable and aggressive, where women with pain felt that their experiences were being doubted by others (Juuso, Skär, Olsson, & Söderberg, 2011). Striving to reach a balance in everyday life to manage the pain was described as a main concern for women with fibromyalgia (Hallberg, & Bergman, 2011).

Coping with chronic pain is seen as important in the rehabilitation of patients with chronic pain. The prevalence of moderate to severe chronic musculoskeletal pain in the Swedish adult population is about 20% and many of the individuals find it difficult to cope with their pain (The Swedish Council on Technology Assessment on Health Care, 2006). The prevalence is related to age, sex, and socioeconomic status (Bergman, 2005); and women are at greater risk than men for developing chronic pain disorders (Fillingim, 2000). Improvement in coping with chronic pain has been reported after treatment with cognitive behavioral therapy, but among the relevant studies there is no explicit inclusion of patients with chronic pain and the difficulties in communicating and cooperating in therapy because of psychiatric co-morbidity (The Swedish council on Technology Assessment on Health Care, 2006). In the rehabilitation of patients with more complex chronic pain disorders, a model for multimodal rehabilitation (MMR) has been developed in Sweden (Gerdele, Stålnacke, Söderlund, & Åsenlöf, 2011). The MMR contains psychological, physical, and pedagogic components and requires that the patients are active and motivated. In a literature review (Gerdele et al., 2011), the effect of MMR is described, and the method has been found to have a positive effect on patients with fibromyalgia, even if not all patients did benefit from the treatment. The authors also highlight the need for additional research in this area.

Cognitive therapy (CT) implies a patient-centered approach (Malterud & Hunskaar, 2002; Willis & Sanders, 2000). In a patient-centered approach, patients’ total situation needs to be taken into account and patients are encouraged to talk about their own beliefs and anxieties about their symptoms. There is a focus on empowerment. This approach also implies that the therapist/physician alternates between the understanding of the patient and the medical disease (D'Elia, 2004). For patients with chronic pain a cognitive behavioral perspective has been considered to be more important than a specific technique (Turk, Swanson, & Tunks, 2008). This approach can combine stress management with problem solving, goal setting, pacing of activities, and assertiveness in order to give the individual tools to deal with a life with chronic pain. The therapeutic model needs to be adjusted to the patient's situation. In CT, mindfulness training has become increasingly important as a tool for the patient to observe and let go of painful thoughts and feelings, and for the acceptance of circumstances one cannot influence. With mindfulness it may be possible to handle pain conditions (Siegel, 2005) and to find ways of self-acceptance (Germer, 2009). In the treatment of patients with chronic pain, support and tools to handle the pain has been reported as important (Steihag et al., 2001). In the training program used in that study, the focus was changed from exercise and education to movement and interaction. Less time was spent on instruction and more on discussions. Instead of reducing pain the aim became to provide tools for the women to handle their pain.

In the rehabilitation, the patient's perspective needs to be acknowledged. It has been argued that the physician needs to understand the patient's disease and illness experiences from the person's perspective (Malterud & Hunskaar, 2002). When the patient's perspective on health and illness is overlooked in health care, such experiences may cause an unnecessary suffering (Berglund, Westin, Svanström, & Sundler, 2012). Malterud and Hollnagel (2007) have presented an awareness model for a patient-centered clinical method where the tasks of the clinician are to identify and pursue both the medical agenda as well as the patient's agenda in clinical encounters. Patients being listened to and confirmed in their experiences of pain can feel recognized when sharing their emotions with members in a group or with a therapist/physician (Steihag, Ahlsen, & Malterud, 2002). This recognition is similar to a little child's interaction with its mother where the child, for example, can express joy of an amusing toy and the mother can recognize and share this feeling. Mutual recognition implies the ability to understand other people's subjective perspective and to get validation of experiences and emotions. According to Bowlby's attachment theory (Bowlby, 1988), this mutual recognition or attunement is important for a secure attachment between the small child and its mother or other caregiver. Hereby a secure base can be formed from where the child is free to explore the surroundings nearby knowing that mother is there if needed. Attachment behavior can be activated in adults in case of, for example, danger or disease. In therapy with insecurely attached patients, a new secure attachment relationship with the therapist may be essential in order to integrate experiences that could not be integrated in an insecure relationship (Wallin, 2007).

Frank (1995) argues that seriously ill people are wounded both in body and voices. There may be needs for patients to tell their stories to construct new maps and to understand their everyday world, in order to handle their illness experiences and feel well. Patients living with chaotic stories probably have a special need for someone who is willing to listen to them even if this may be a painful experience for both participants. Research is needed on the treatment of patients with chronic pain disorders. Methods are required to improve these patients’ quality of life and function in daily life. Therefore, the aim of this study was to explore the meaning of attachment and mindfulness-based CT among patients with chronic pain and psychiatric co-morbidity, who had not benefited from conventional pain treatment.

## Methods

A phenomenological approach within a lifeworld perspective was used (Dahlberg, Dahlberg, & Nyström, 2008). Lifeworld-based research is focused on the world as it is experienced. Practicing this approach involves an open and sensitive attitude to the studied phenomenon and to the complexity of the lifeworld. Thereby it was possible to reveal the patients’ subjective experience of illness and experiences of the therapeutic process. The subjective experiences of body, health, and existence must be taken into consideration in order to understand the experiences of illness and disease of another human being (Toombs, 1992).

### Participants

Ten patients were interviewed after they had completed therapy. Twenty patients with chronic pain referred for psychiatric assessment were offered treatment with CT (18 women and 2 men). Of those, 13 patients completed the therapy, one of those patients declined recording, one patient did not turn up at the interview, and one recording failed, resulting in 10 recorded interviews (nine women and one man). Those who did not complete the therapy only attended therapy session a few times, and were therefore not interviewed. The 10 patients interviewed were diagnosed with: Fibromyalgia (1), chronic wide spread pain (3), whiplash trauma (5), and chronic regional pain (1). Additionally, a psychiatric diagnosis according to DSM IV was present in these patients and the most common diagnoses were anxiety, mixed anxiety and depression, and maladaptive stress reaction. Earlier traumatic experiences were found in seven of the patients. The patients were aged between 32 and 60 years, with a mean age of 44, and all but one who worked 25% were on full-time sick-leave.

### The therapy

The duration of the therapy was 7–13 months with individual sessions every second week as a rule. The therapy was carried out by the therapist/physician. The therapy focused on the patients’ experiences. In the therapy the patients were encouraged to narrate their life stories and their illness experiences. The physician/therapist listened attentively and when needed confirmed their stories in order to legitimize the patients’ feelings and thoughts about his/her illness. By means of open and Socratic questions the patients were guided to discover new perspectives and to restructure his/her thoughts (D'Elia, 2004). An active collaboration between the patient and the therapist was important. For all patients in this study the therapy sessions also involved mindfulness training. This meant to learn to let go of stress and negative thoughts when appearing, and by focusing on breathing and being present in the moment with acceptance (Ecclestone & Crombez, 2007; Kroese, 2002). Equally important was the therapist's ability to be present and attentive to both one's own feelings as well as the patient's. This attunement may facilitate the patient's experience of a secure base in the therapy (Bowlby, 1988; Wallin, 2007).

### Interviews

Qualitative semi-structured interviews were used for data gathering (Dahlberg et al., 2008). The therapies were carried out by BP and the interviews afterwards were conducted by AS. A semi-structured interview guide was used and the patients were informed about the questions in the interview guide before the interview started. The guide was used only as a support in the interview. The patients could also choose the order of answering the questions. The questions concerned their current life situation and their experiences of change in terms of psychological health, experiences of pain, relationships to other people, and their ability to carry out daily activities. One question dealt with the reactions of significant others. What did they experience from family members? There were also questions about the therapy: What were their expectations before the therapy? How was the collaboration with the therapist? What was important for progress in therapy? What were their thoughts about the future and the effect of therapy in the long-term perspective? The interviews were recorded and the duration of the interviews was between 1 and 1.5 h. The interviews were transcribed verbatim.

### Data analysis

A phenomenological analysis with a focus on meanings was performed (Dahlberg et al., 2008). The analysis was mainly performed by three of the authors (BP, AS, and AJS). In the initial phase, the text was carefully read through a number of times with an open mind to get a naïve understanding and a sense of the whole. Clusters or temporary patterns of meanings appeared in the text. The analysis can be described as a movement between the whole, the parts, and the whole. After the naïve reading, the text was divided into meaning units. This was carried out with respect to the meanings found in the analysis. The analysis was also guided by the method for narrative configuration described by Polkinghorne (1995). Meanings found were related to narratives in the interviews, and various aspects of lived experiences of pain and therapy were used to illustrate the meanings described in the constituents. In the analysis it was essential that the researchers adopted a phenomenological attitude restraining their pre-understanding from forcing a meaning to appear. The reflective lifeworld approach used in this study calls attention to principles of openness and “bridling,” which means that as researchers we must critically reflect on the research process and our understanding of the phenomenon (Dahlberg et al., 2008). The analysis was discussed several times among the researchers, and we tried to question our pre-understanding to minimize its influence on the emerging meanings. The varied meanings formed a pattern from which the essence of the phenomenon could be distinguished at a more abstract and general level. The essence also binds the meanings together, and it is presented in the first part of the findings below together with its three meaning constituents. Quotes and narratives are used to illustrate the meanings described in the constituents and the essence of the phenomenon. In the narratives the participants were given fictitious names in order to protect their identity. Examples from some participants are given in the text, even if the meanings occurred in more than one interview.

### Ethical approval

The ethics committee of Göteborg University approved the studies (032-06). We have complied with standards for the ethical conduct of research, including confidentiality and anonymity, and the study conforms to the Declaration of Helsinki. The participants filled in an informed consent before the start of the therapy, and their participation in the interview was voluntary and did not affect their future treatment or follow-up. After the interview, a further follow-up with the therapist was a rule.

## Results

The essential meaning of participating in the therapy was an experience of creating meaning in life with chronic pain. In the interaction with the therapist the patients could gain trust and put words on difficult experiences and strange feelings, as well as to get an understanding of one's own situation. The therapy became a starting point for changes and managing life with pain in a meaningful way. In the therapeutic sessions the patients had an experience of being listened to and of being acknowledged. Depending on the individual's situation and circumstances, different strategies to handle and manage everyday life were found with support and trust from the therapist. To manage life with pain was hard but possible when there was a will to change. In the therapy the past was linked to the present and a movement toward something new could start. Living with chronic pain had been an experience of chaos. Before the therapy the patients experienced that they had reached a limit where they had no more strength to go on although they also had a desire to feel better. During the therapy the patients could find meaning in life with pain. The therapy and the process of interaction with the therapist were experienced as meaningful for the patients’ well-being and the management of their pain. The phenomenon and its essence is illustrated by its three meaning constituents: finding meaning in life with pain; feeling empowered when learning to let go and leave things behind; and building an understanding of one's body and symptoms.

### Finding meaning in life with pain

Living with chronic pain had negative consequences for the patients’ everyday life. When not being able to manage the pain, the patients gradually lost earlier roles and identity, and their pain had restricted their daily living. The experience of pain was something existential involving feelings and emotions difficult to manage and to express. In different ways the patients experienced the therapy as a way back to life. They could go from earlier chaos and despair back to a life they could cope with. By opening up for one's own feelings and to other persons, negative experiences and stressful thoughts could change. This was experienced as important in the patients’ way back to a more meaningful life.

Anne was born of a young mother and grew up with her grandparents. She always had high demands on herself. After a car accident 1999 when she was in her thirties she suffered from a whiplash trauma with persistent severe symptoms leading to disability pension. In the interview Anne described a way from having no foothold and being in the dark towards retrieving her previous roles as a mother, a wife, and a professional.

There was grief when I lost my roles, my professional role. I feel like a failure. I can't cope with that either. Somewhere inside you feel ashamed.

She had long been a victim of her feelings, lacking tools to handle them. During the therapy she was able to narrate the story of her life, and in connection with her past experiences, her present feelings about not being able to handle her problems were more understandable for her.

I reacted strongly to certain situations, triggered by things that happened in my childhood. And I could see the associations between what happened and the way I have handled my life and my feelings. It was nothing wrong. I was brought up to be an independent person.

Anne had a motivation and desire to find a way back to her previous life. She had many of the answers about how to do it but not the ability.

I had the answer how to do it but I could not take hold of it myself … I needed someone who pushed me in the right direction.

And she began to feel free to open up also outside the therapy room to herself and to others.

At first it was very hard work, like throwing away your mask. I can now put words on feeling abandoned and deserted. My husband prefers that to just snubbing and hissing.

John's life was also changed in a car accident more than 10 years ago. He was then 24 years old. Like Anne, he had a strong desire to get back to life as it was before the accident but in his case he gradually had to accept that it would not be the same and he needed to put words on these feelings. In doing so, he also had to work with his traumatic memories of the car accident when his car got hit by another car running out of control in bad road conditions. He had to handle his anxiety when driving or going by car.

I see now that I will never get rid of my pain … it can be less or more. It is important to be allowed to talk and that you feel someone is listening. It is nothing odd to come here. It is, so to say, part of my health care and part of my way back. We have talked about focusing and breathing in order not to panic when travelling by car and sitting tensely on guard.

John experienced pain as something restraining his freedom of movement. Pain was an obstacle that forced him over and over again to change direction.

Like before I didn't know the limits of my body … and you were … you were active and then you felt frightfully ill and in pain perhaps a whole week. … You don't have to do that.

Also John found himself more open to his own feelings and towards other people. Instead of getting angry and building up tension inside his body it was easier to open up and communicate his feelings.

If someone just touches you with a cart when shopping you get angry and there is a misunderstanding. I have learned to stop and think a little. I can tell people, but more calmly.

### Feeling empowered when learning to let go and leave things behind

Before the therapy the patients had experienced that their former strategies to handle life had not worked, having difficulties to cope with their pain and problems. That made them feel confused and powerless. Before the therapy they had not been able to handle their situation, and the pain had restricted their life in various negative ways. In the therapy they were able to put words on their experiences, thoughts, and feelings. The interaction with the therapist was experienced as a secure base. Experiencing a mutual relation with the therapist seemed to give them strength and motivation. They were then able to find strategies to change their old patterns and to try something new. Leaving old things behind made their burden lesser. Mary experienced herself as captured in a spiral motion of pain, stress, and exhaustion; working with mentally retarded children. In leisure hours she always had been very active at home and helping others. After taking care of her parents before they died she felt exhausted and her brothers and sisters even accused her of being unfair and greedy. Still she went on working with disabled children until after a year or two she could not manage her pain in her back, arms, and neck. And she felt she was in need of help. “I told my doctor that I had to be referred for treatment with CT. He would not agree to that at first but I stood up for myself.” Until then she had not paid attention to herself. Her focus had been on the needs and wishes of other people. Earlier it was natural for her to finish her work and not leave anything for tomorrow but now she had learned to think in a different way.

Yes it is like a huge backpack and you can unload some bricks here and there … you leave them and it becomes much easier. You don't have to … I don't have to. It is a nice feeling. But now I think I don't have to do it today and maybe not even tomorrow.

Trying these new strategies gave her new hope and joy.

Those meetings with her, I think we have fun. It has given me something. My pain is still there but I can get rid of this stress and the heavy weight on my back and the whole body, there is a light ahead.

In some patients, the pain along with illness and traumatic memories from childhood or later in life has disturbed the balance and natural flow of life, for example, feeling rigid or immobile. The patients experienced being strengthened when body and mind as well as past and present was integrated. There was a need of setting one's account and to accept life as it had become, which became possible in the therapy. From that base, the patients could view themselves in a new way and from there go on with their lives.

Christine, a married woman 60 years old, had traumatic memories of her childhood and as a teenager, and she realized that those memories still affected her.

I saw a person … being stuck in a certain way of thinking … yes … and I think that after that I have like started trying to look at myself in another way. I think that I have been self-destructive … yes … having a need to oppress … to push myself down in a way.

In the therapy, she could feel a normalization of her earlier experiences and that it was safe to test new strategies leaving the old things behind.

In a way I think that the right questions are important in order to be able to move on. She has played things down … like everybody sometimes faces difficulties. And it will be no catastrophe if I leave the old things behind, even if it is sort of a safety. I must dare to try something new.

In addition to chronic pain, Christine also needed treatment for Parkinson's disease, and a moderate depression. There was a need for a comprehensive view of her situation and she experienced a new physical and mental flexibility and movement in her life.

BP saw that I was depressed and wanted me to start medication … and she has said the whole time that I should start going for a walk … many had said so and I thought I had no energy … but when I started this medication I suddenly had the energy for walking.

### Building an understanding of one's body and symptoms

When the patients experienced that they could understand their body and symptoms in a meaningful way they could start a journey where to go from chaos to comprehensibility and manageability. Some of the patients had a varying degree of burnout in addition to their chronic pain. This often brought about a variety of bodily symptoms as well as bodily and mental exhaustion and anxiety. Their symptoms had become overwhelming and there was a need of explanation and to sort things out in order to manage their pain and to live a meaningful life. For some patients, their understanding of somatic symptoms was an important part in the therapeutic process.

Kate is in her late forties and is a mother of four children. Her youngest son is autistic, and two of the other children, like herself, suffer from multiple sclerosis. She had a traumatic childhood and always took responsibility for her brothers and sisters and now for her own family. A few years ago she was burnt out and lost control of her life. In addition to wide spread pain she was mentally and bodily exhausted. Before that, during her whole life, she had not taken notice of her bodily symptoms and not allowed herself to rest when she was tired.

I lost my foothold and sense of reality … lost everything. I didn't think it would happen to me. I am used to always keep going … Everything happened all at once … millions of symptoms.

Gradually Kate was able to get knowledge of her symptoms and sort them out and thereby started a process of normalization and acceptance. In the therapy, the control of her life was regained when being able to understand her body and symptoms.

Thanks to her [the therapist] … such as … everything was straightened out. I can fit in all the facts … as to speak … so that there is nothing that suddenly appears … because then I lose control immediately … and that can't happen. I must be able to live a fairly good life anyway … as normal as possible with all symptoms in my body. I must face it, read about it and understand it. I feel good about coming here. Nothing has been negative and I haven't been told I am an idiot.

She described that she has become more observant of signals from her body in her daily life vis-á-vis herself and others.

I don't have to go down to the basement when it is dark. I can do it the following day when it is light for example. I work with alternatives.

Helen talked about her experiences of anxiety that had increased after she had to stop taking Tramadol, and her difficult social situation living with an alcoholic, her child's father. Her pain had started after a whiplash trauma 10 years earlier when she was in her thirties. When she started in therapy her anxiety and panic attacks prevented her from living a normal life.

I didn't dare go out for a walk or something … was afraid of collapsing. I wanted to talk to someone who knew about these symptoms and who could explain.

In the therapy, Helen was able to talk about her anxiety and to get explanations and investigations of bodily symptoms. As she eventually felt safer and stronger, she was motivated to fight for a better life.

And I have been sitting here saying the same thing tens of times: Can you die from it? At first she told me that she did not think it was serious but she always checked it up in order to reassure me and I was satisfied with that. I had to do something myself in order to get better. But I have had to fight for more than a year and a half, but now it is worth it.

When Helen managed to move with her child, away from the child's father, to her old apartment, she experienced a gradual improvement of her health. She then started to hope for a normal life even if she experienced a lack of trust in close relations.

Yes, we could go to bed without feeling afraid of him coming in and waking us up. The physical pain is not as painful as the pain in your soul. I think that I am more healthy now … and I could manage a half time job.

## Discussion

The CT used in the current study was found to be meaningful for the patients in everyday life and their well-being. The patients described it as meaningful to be seen and listened to, and that there was time for them to tell their story. In the narratives the patients’ experiences from the past were linked with the present and transformed into a new understanding. There was also a need for time and to find a secure base in the therapy from where to go from old to new strategies. Earlier these patients had not benefited from conventional pain treatment. In this study, the therapy was adjusted to their special needs, and led by a trained therapist and physician specialized in treating patients with chronic pain. The therapy used in this study was experienced as meaningful for the patients in finding ways to manage life with pain.

### Creating a meaning in life with chronic pain

In the present study many of the patients felt a lack of control and being in a chaotic situation because of lack of explanation of the origin of pain and other physical symptoms. They had a need to tell their story in order to handle the situation. In the literature, narrative construction can be a way for people to feel well in illness (Frank, 1995). In this study, the therapy facilitated their stories to be told. Especially in the beginning of the therapy time was spent on listening to and explaining these symptoms as well as referring for additional investigations when needed. Merleau-Ponty (1995/1945) emphasize that understanding of illness needs to begin with an understanding of lived experiences. When a patient tells the story of her/his life with pain and illness both the life of the teller and of the listener who enters the space of the story are effected, and Frank (1995) describes this as: “The self is understood as coming to be human in relation to the other” (p. 15). This creation of meaning and also finding explanations and new strategies have much in common with the characteristics of the Sense of Coherence (SOC) theory (Antonovsky, 1987), where a person with a strong SOC is able to find her/his life meaningful, comprehensible, and manageable. Polkinghorne (1991) described a narrative as a cognitive process that gives meaning to temporal events by identifying them as parts of a plot. Thus events are organized into various stories about the self, creating self-understanding and providing answers to the question “Who am I?” When the patient's perspective of health and illness is overlooked it can cause unnecessary suffering (Berglund et al., 2012).

### Time for storytelling – a way to build trust and a secure base in therapy

The results revealed that patients with traumatic experiences might be very incoherent in their narratives and they needed time to gain trust. The therapist initially had a role of holding and normalizing feelings and symptoms. A majority of the patients had an insecure attachment pattern and many had traumatic experiences in childhood or as adults. This fact implied demands on the therapist to try to create a secure base in therapy from where the patient could feel safe to try new strategies, just like the small child exploring the world with its mother as a secure base (Bowlby, 1988). An adult insecure attachment pattern both represents a risk factor for developing chronic pain and a vulnerability factor for poor outcome (Meredith, Strong, & Feeney, 2007). In this study the therapy was experienced as a secure base that could strengthen and empower the patients. The patients in the current study had not improved with traditional rehabilitation for chronic pain and only 13 out of 20 patients had the motivation and ability to go through with the therapy.

### The importance of mindfulness and acceptance

For all patients the therapy sessions also involved mindfulness training to learn to let go of stress and negative thoughts by focusing on breathing and presence in the moment. Hereby it can be possible to become an observer instead of a victim (Kroese, 2002). In the present study, patients experienced being able to view themselves in a new way, facilitating their ability to handle life with pain. Moreover mindfulness has been shown to enhance acceptance of pain related symptoms and to ameliorate psycho-social well-being (McCracken & Gauntlett-Gilbert, 2007). Mindfulness also seems to be essential in the therapeutic relation. Being in the present moment facilitates a meeting between the patient and the therapist (Stern, 2004). The interaction between the patient and therapist is important in the forming of a secure base (Schore, 2003). Tang, Salkovskis, and Hanna (2007) have described mental defeat in trauma and also in chronic pain. This notion applied especially to patients seeking help and treatment for their problem. The treatment recommended in that study was mindfulness, compassion, and “the healing of self.” This kind of holding and stress reduction was of great use with most of the patients in the present study, especially in the beginning of the therapy.

### The need for time in the process of motivation and change

In the beginning of the therapy several patients reported that they had reached a limit where they had no strategies or means of handling their situation. They described a loss of power and energy. Former strategies to act and to achieve no longer worked. Those patients who left therapy prematurely mostly had lacked motivation and energy. Patients who carried through with their therapy had a need for time to make a change at their own rate. It was a question of holding and timing where the patients could feel safe and take their time to find the power and motivation for change. Similar to results in our study, Steihag et al. (2001) have pointed out the importance of finding time enough to start up a process of change in the treatment of patients with chronic pain. In their study, discussions were found to facilitate the patients’ understanding of their situation and their symptoms. Prochaska and DiClemente (1986) have identified specific stages of change. They described a change process in which patients in the first stage, like many of our patients, lack power and motivation to find a solution to their problems and how, during a period of ambivalence, they begin to see and contemplate trying alternative solutions. According to Prochaska and DiClemente there is a need for time in this phase, which is also in accordance with our findings. Some patients were able to sense continuity between the past and the possibility and novelty of the future (Todres, Galvin, & Holloway, 2009).

### Strengths and limitations

The outcome of this study elucidates the experiences of this special group of patients with chronic pain and psychiatric co-morbidity. The strength of this study is that there is not much research done on this special group of patients with chronic pain and the results are supported by existing knowledge about treatment of patients with trauma and vulnerability. All patients did not complete the therapy. Of those who did, 12 were interviewed and 10 of these interviews were recorded. The interviews were rich in meanings and a phenomenological approach was found to be well suited. The analysis was performed by researchers with a multi-professional background with a broad experience of chronic pain and/or qualitative research, a fact which might have contributed to a holistic approach. In the interview group 9 of 10 were women, a gender distribution that is not uncommon in chronic pain patients (Fillingim, 2000). In the initial therapy group, 7 out of 20 left therapy mainly because of psycho-social stress and/or lack of energy and motivation. The fact that as many as seven patients did not complete the therapeutic sessions might be explained by vulnerability factors such as trauma and an insecure attachment at least to some extent. An issue for future research would be to apply this therapeutic method to new groups of patients with chronic pain and psychiatric co-morbidity. Also, those who leave the therapy prematurely should have an opportunity to participate in an interview in order to find factors contributing to therapy failure.

### Clinical implication

The patients in the current study had not benefited from conventional pain treatment. The primary aim of the therapy was to improve their well-being and ability to cope with their symptoms. The therapy was adjusted to the patients’ special needs. In line with Malterud and Hunskaar (2002) we argue that a patient-centered approach is important in the health care of patients with chronic pain. A trained psychotherapist with an expert knowledge of patients with chronic pain might be required. These patients often have difficulties in finding enough support and understanding in primary health care and in psychiatric settings. Our study also indicated that there is a need for time in order to allow the patients to gain trust and to make changes at her/his own pace. It was then possible for patients to improve their well-being and a few of them went back to work to some extent. Thus CT with focus on attachment and mindfulness seems to be of value for these patients. Several of the patients such as Kate and Helen have also pointed out the importance of getting explanations and an investigation of somatic symptoms. In the health care of patients with chronic pain there needs to be a multimodal team with expert knowledge of both somatic and psychological aspects of pain. Offering therapy to these patients is of value, even if it is expensive and the patients might not go back to work.

## Conclusion

The study concluded that the therapy and the process of interaction with the therapist were meaningful for the patients’ well-being and for a better management of pain. During the therapy the patients were able to initiate a movement of change. In the current group of patients, mainly women, life with chronic pain was experienced as a chaos and a loss of control and integrity. Many had reached their limits where they had no more strength to go on and old strategies were unsuccessful. In this phase it was important to them to feel that there was someone who listened to them, confirmed the legitimacy of their experiences, and that there was time to gain trust. For those who had strength and motivation, the therapy started a movement of change and this process looked somewhat different for each individual. There was a change in attitude towards oneself, pain, and relations to other people; meaningful for their well-being in everyday life. The CT used in this study was adjusted to the patients’ special needs and a trained psychotherapist with a special knowledge of treatment of patients with chronic pain might be required.
